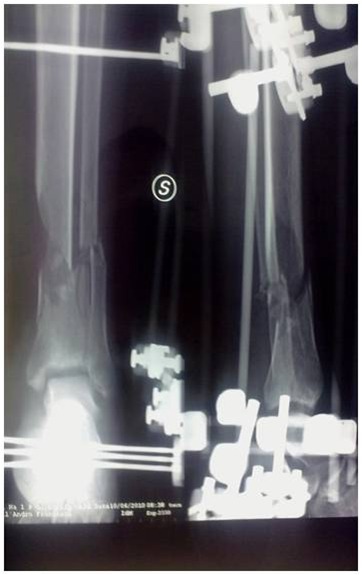# Teriparatide in Fracture Non-Unions

**Published:** 2014-09-01

**Authors:** C. Coppola, A. Del Buono, N. Maffulli

**Affiliations:** 1 Department of Orthopaedic and Trauma Surgery, Hospital “S. Maria di Loreto Nuovo”, Naples, Italy.; 2 Department of Orthopaedics and Traumatology, Sant’Anna Hospital, Via Ravona, San Fermo della Battaglia (Como), Italy.; 3 Department of Musculoskeletal Disorders, Faculty of Medicine and Surgery, University of Salerno, Salerno, Italy ; Centre for Sports and Exercise Medicine; 4 Barts and The London School of Medicine and Dentistry, Mile End Hospital, 275 Bancroft Road, London E1 4DG, England.

**Keywords:** Fractures, non-union, teriparatide, surgery, internal fixation

## Abstract

**Background:**

The use of teriparatide in the management of fracture disorders is poorly documented. This study aims to show that teriparatide administration may improve the healing process in patients with nonunions after open fixation of traumatic fractures of the lower limb.

**Methods:**

Four patients received Teriparatide for management of non-unions after open fixation of traumatic fractures of the lower limb.

**Results:**

Teriparatide administration resulted in adequate bone callus over the site of nonunion in all the patients, and clinical and radiographic evidence of sound union.

**Conclusions:**

The efficacy of teriparatide in delayed or non unions is still unclear. It may induce an angiogenetic response which counteracts the features responsible for development of non-union.

**Level of Evidence:**

Level IV, therapeutic case series.

## Background

Approximately 5–10% of bone fractures do not heal promptly, and require further treatment^1^. Advanced therapies for fracture healing are increasingly emerging, but it is crucial to assess their clinical effects^2^. By stimulating osteoblasts and reducing osteoblast apoptosis, intermittent administration of human parathyroid hormone (PTH) increases callus formation, improves mechanical strength^3^, and results in increased osteoblast life span^4^. PTH may increase cancellous bone formation by stimulating osteoblasts^5^, with unclear effects on the periosteal surface, and decreased risks for vertebral and nonvertebral fractures after a median treatment of 19 months^6^. Human PTH stimulates osteo-progenitor cells to proliferate early, differentiate, and produce bone matrix proteins, mostly after 1–2 weeks of therapy^7^. In rats, human PTH enhances fracture healing, increases mechanical and histological properties^3^, and promotes the integration of orthopedic implants into the bone^8^, improving bone in growth and pullout strength^9^. In management of osteoporosis, compared to anti-resorptive drugs, Teriparatide, recombinant human PTH(1–34), acts as bone anabolic agent^10^ on spinal bone mass^11^, restores microarchitecture and trabecular connectivity, enhances cortical thickness^12^, and improves bone strength, mostly in bones rich of cancellous bone^6^. Administered once daily through subcutaneous self-injection, it results in a rapid and greater increase in vertebral bone mineral density, decreases risk of vertebral and non-vertebral fractures in postmenopausal women and men with osteoporosis, and provides encouraging pre-clinical results in fracture healing^13^. Although relatively few data have been published on the administration of Teriparatide for management of fracture and related complication in humans, and no definitive conclusions around its effectiveness may be drawn, a once daily subcutaneous injection could enhance fracture-healing in humans^14,15^.

We report on four patients who received Teriparatide for management of nonunions after open fixation of traumatic fractures of the lower limb.

## Methods

### Patient 1

**Figure 1:**
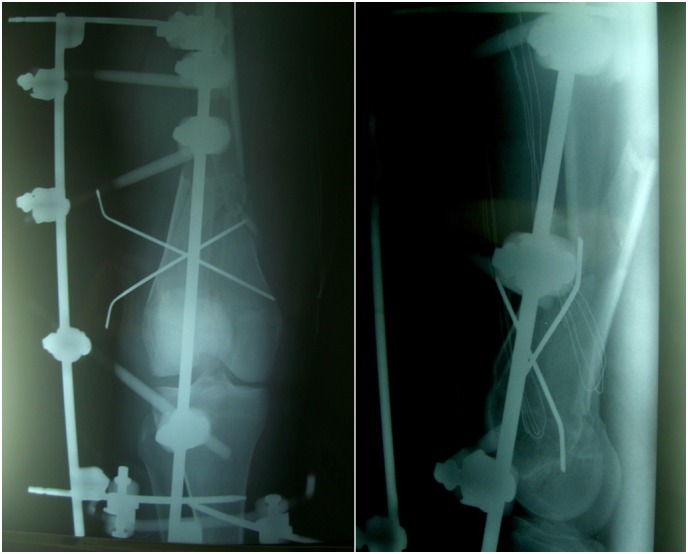


**Figure 2:**
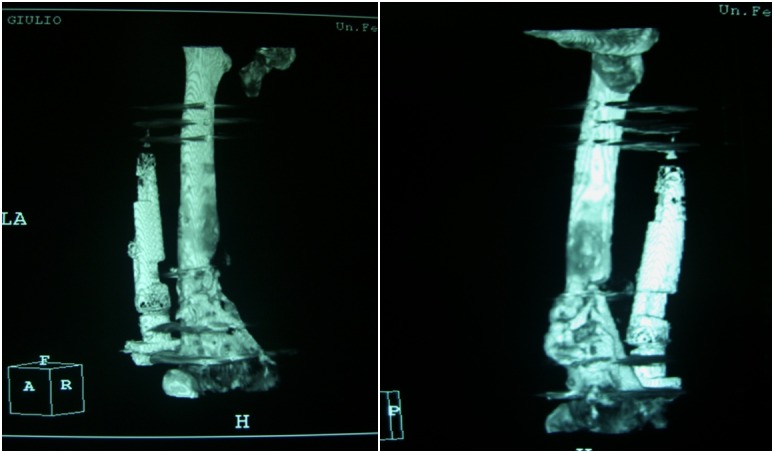


**Figure 3:**
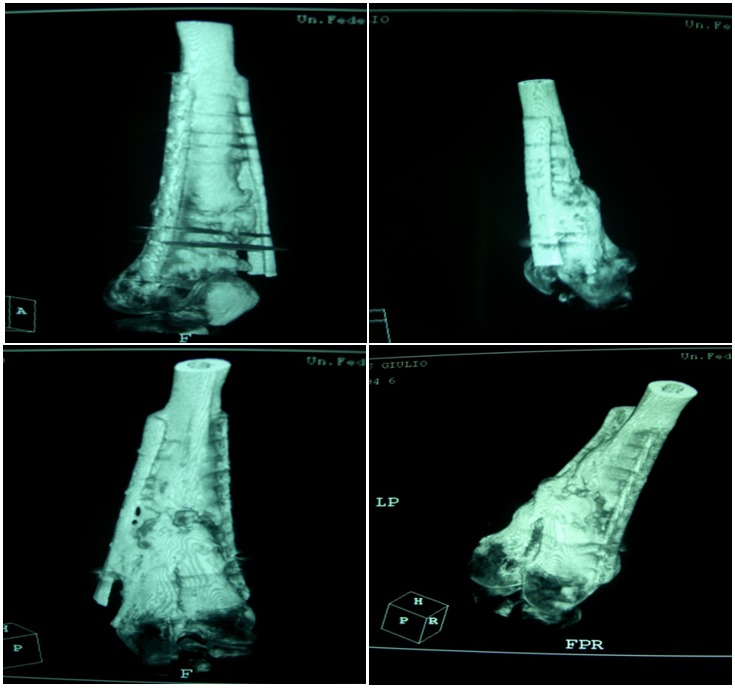


A 36-year-old male healthy swimmer, nonsmoking sustained a Gustilo III 3B fracture^16^ of the right femur, 33A3.1 according to the AO classification system, in a road accident (Fig 1). He underwent open reduction and mono axial external fixation supplemented by Kirschner wires (Fig 1). At four weeks, after the soft tissues had healed, an external fixator was applied. Imaging two months after surgery showed poor bone apposition in the medial and posterior aspects of the site of fracture (Fig 2). Four months after the initial injury, the distal external fixation pins had loosened and there was delay in healing. The patient underwent open reduction, fixation and stabilization of the fracture with a mono axial fixator supplemented with Allomatrix (Wright Medical Technology, Inc, Arlington, Tenn)^17,18^. Eleven months after the trauma, despite signs of nonunion at radiographic evaluation, the external fixator was removed, and the patient started to walk in a protected brace. Laboratory investigations, including serum alkaline phosphatase, PTH, calcium, creatinine, and 25 (OH) vitamin D were normal, excluding any metabolic disorder. After 15 months from the original trauma, the patient underwent open reduction and internal fixation with a condylar plate, application of platelet rich plasma and implantation of bone allograft. At 20 months, given the poor bone integration of the graft and the absence of bone callus, the patient started treatment with subctunaneous injection of teriparatide (1 injection of 20 μg daily), calcium and vitamin D. After 9 months of treatment, the 3D CT showed complete integration around the plate and the bone graft, with adequate formation of bone callus at the site of nonunion (Fig 3). The serum levels of alkaline phosphatase, increased during the 9 months of therapy with teriparatide, normalised within 3 months of interruption of teriparatide administration. At follow up 3 and 5 years from the last operation, the nonunion was healed and the patient was satisfied in terms of daily and sport activity. Clinically, a 3.5 cm discrepancy was well tolerated and balanced by wearing a shoe with a raise and an insole.

### Patient 2

**Figure 4:**
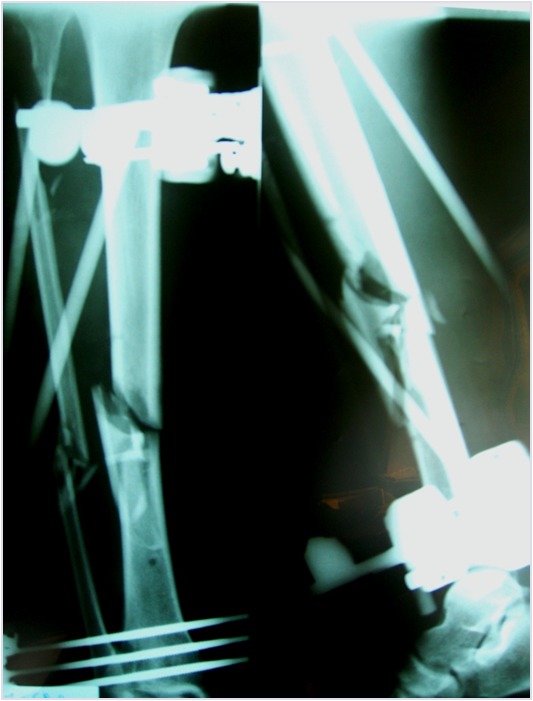


A 33-year-old healthy non-smoking male underwent reduction and external fixation of a post-traumatic (traffic accident) Gustilo III 3B fracture of the right tibia and fibula, 42A.2 according to the AO classification (Fig 4). At 5 months from surgery, given the absence of any signs of healing on radiographs, the patient underwent open reduction and reamed intramedullary nailing. At 5 months from the nailing, the patient continued to report pain, and was only able to partially weight bear with two elbow crutches. Radiographs showed lack of evident callus, and atrophic nonunion.

**Figure 5:**
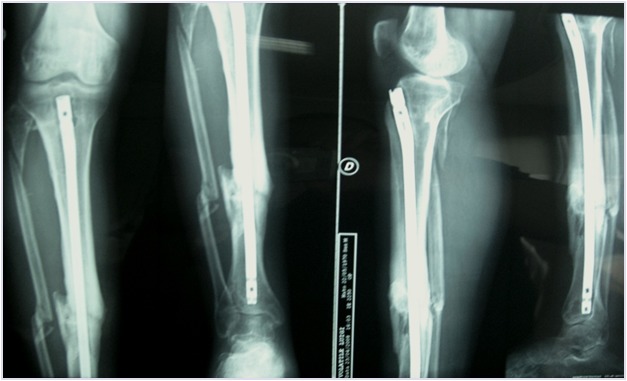


The patient started treatment with teriparatide (20 μg subcutaneous injection daily), calcium and vitamin D. After 4 months of teriparatide administration, radiographs showed good integration of the bone around the nail, and adequate bone callus over the site of nonunion (Fig 5). Ten months later, he returned to his pre-operative occupation of insurance salesman and sport activities (modern pentathlon).

### Patient 3

**Figure 6:**
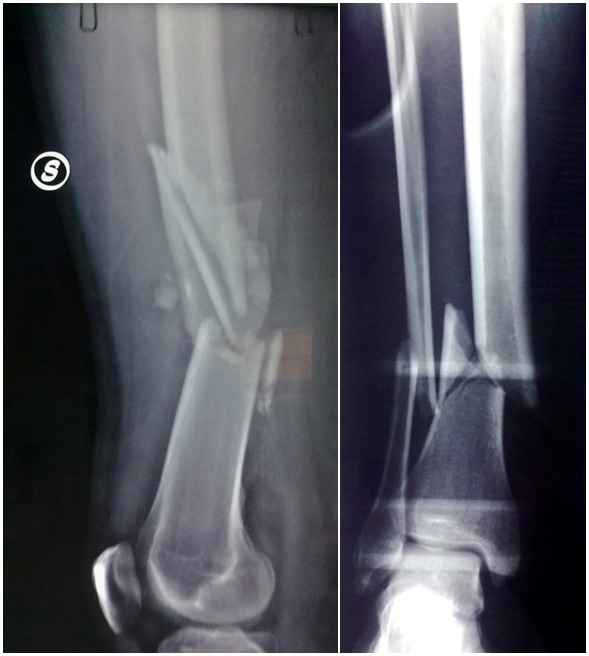


**Figure 7:**
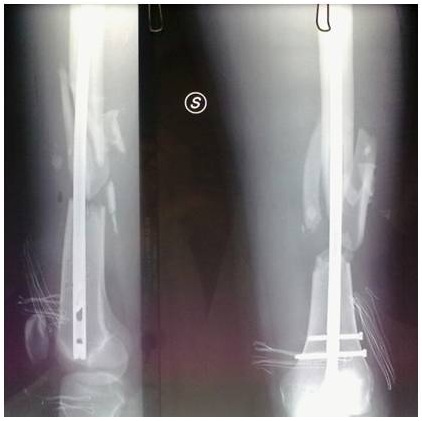


A 28-year-old healthy male nonsmoking truck driver sustained a Gustilo III 3B fracture of the left femoral shaft (32B.2 by AO classification) and left tibia and fibula (43A.3 by AO classification) (Fig 6) after a road accident. He underwent reduction and internal fixation using a reamed retrograde femoral nail, and external fixation of the fracture of the tibia and fibula. At 4 months, radiographs showed poor healing, lack of bone callus, and atrophic nonunion for both fractures (Fig 7).

**Figure 8:**
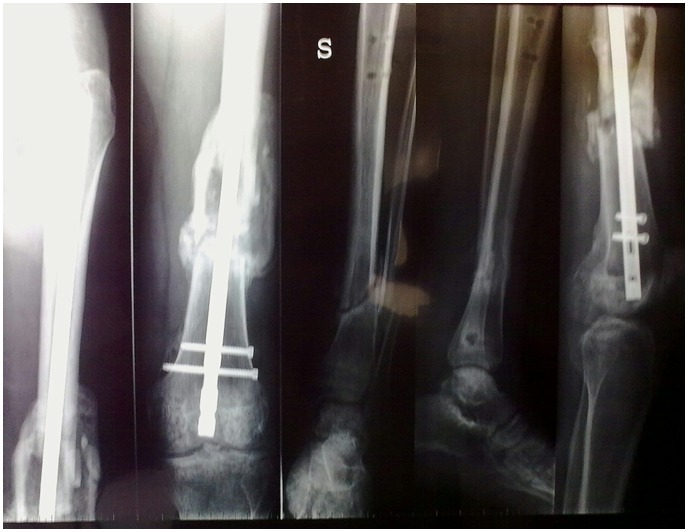


The patient underwent removal of the external fixator of the lower leg, and started treatment with teriparatide (20 μg subcutaneous injection daily), calcium and vitamin D. After 3 months of therapy, radiographs showed good integration of the bone around the nail, and adequate callus over the site of nonunion (Fig 8). Twelve months later, he started to work again and had changed sport (body building). Flexion of the knee was to 95°, extension was full.

### Patient 4

**Figure 9:**
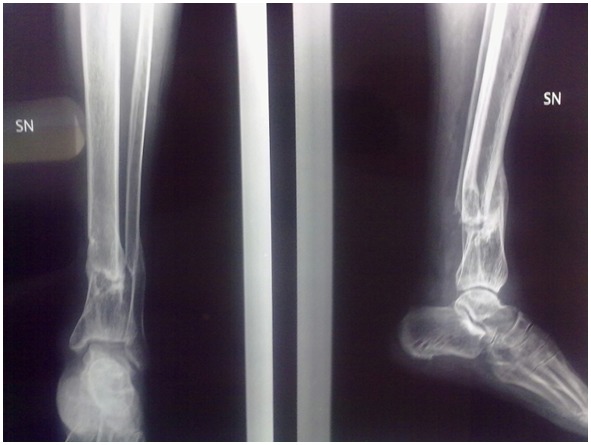


A 30-year-old healthy, engineer, nonsmoking male underwent reduction and external fixation (Hoffmann II device) of 43A.3 (by AO classification) Gustilo III 3B fracture of the left tibia and fibula. At 4 months, radiographs did not show any signs of bone healing (Fig 9). At that time, the patient underwent removal of the external fixator and administration of teriparatide (20 μg subcutaneous injection daily), calcium and vitamin D. Given the soft tissue impairment, he could only undergo hyperbaric therapy and plastic surgical procedures. After 4 months of therapy, radiographs showed good integration of the bone, and adequate bone callus over the site of nonunion (Fig 10). Eight months later, he could work and swim. Ankle extension was 10°, flexion 20°.

## Discussion

The management of open fractures of the tibia and femur is challenging. Advanced age, diabetes, corticosteroid treatment, osteoporosis, mechanical and anatomical factors predispose to delayed or impaired union of fractures^19–24^. Undiagnosed metabolic or endocrine disorders of calcium, vitamin D, and parathyroid hormone (PTH) impair fracture healing, and are considered predisposing factors in up to 85% of nonunions^25^. Internal and external fixation can be used for appropriate fracture management, with no definite consensus. Autologous bone grafting is the current gold standard to aid healing in atrophic non-unions^26^, but postoperative pain, infection, nerve or vascular injuries, and donor site discomfort may complicate this procedure^26^. Allogenic grafts eliminate donor morbidity, but may induce immunological sensitization^27^. BMPs induce bone regeneration and promote repair process^26^, but their exact role with respect to type, dose, and carrier, together with their cost-effectiveness, need further clinical delineation^28^. Bone is a dynamic tissue, and PTH regulates bone metabolism and calcium homeostasis in both intra and extracellular fluids, with direct effects on osteoblasts and stromal cells, and indirect activation of osteoclasts^29^. Since human recombinant PTH, teriparatide, reduces the risk of non-vertebral fragility fractures for 18–30 months after discontinuation of treatment, intermittent exposures to human PTH (less than 2 h daily) are increasingly used to improve and accelerate fracture healing, and enhance bone formation in different clinical settings such as the early postoperative period after osteosynthesis and joint replacement^29^. As teriparatide is expensive, its use at the moment should be limited to selected patients presenting severe forms of osteoporosis, presence or history of one or more fractures, exposed high risk for subsequent fractures, or to patients with osteoporosis that have unsatisfactory responses to or intolerance of other osteoporosis therapies^30^. Given its favorable tolerance and treatment compliance, we used teriparatide to treat nonunions after surgical management of open fractures of the lower limb, with good results in terms of pain relief and imaging outcomes. Primarily used for management of severe post-menopausal osteoporotic fractures, teriparatide has been firstly administered 7 weeks after intramedullary nailing in a patient with fractures of the right tibia and fibula^31^.

The therapy improved clinical and radiographic outcomes of the fracture, and the patient returned to run within 3 months. In a series of 34 patients undergoing fracture surgery, teriparatide accelerated healing and allowed early return to normal function^32^. A prospective, randomized, double blind study of 102 postmenopausal women who had undergone conservative management of distal radial fractures showed shorter time to healing after administration of teriparatide (20 mg)^33^. Although the presence of confounding factors does not allow to draw consistent conclusions about the role of teriparatide in fracture healing, teriparatide may accelerate this process. To the best of our knowledge, this is the first case series reporting on the use of teriparatide for management of nou-union in fractures of the lower limb. Chintamaneni et al.^15^ described the first case of sternal fracture nonunion responding to treatment with teriparatide, with dramatic radiographic healing more than 6 months after the initial fracture. Another patient with delayed union of a humeral shaft fracture healed after 5 months of therapy with teriparatide (rhPTH 1–34), without other interventions^14^. A recent prospective randomized controlled study has shown that the additional administration of PTH 1–84 (once-daily injection of 100 μg) significantly improves functional outcomes and fracture healing in women with postmenopausal pelvic fractures compared to control women undergoing calcium and vitamin D administration only^34^.

Side effects of teriparatide include dizziness and leg cramps; hypercalcemia is uncommon, and easily managed modifying the intake of calcium and vitamin D3^35^. Pre-clinical studies on rats exposed to high doses of teriparatide, from 3 to 58 times the approved dose for humans, showed an increased occurrence of osteosarcoma^35^, and teriparatide is contraindicated in patients with Paget disease of bone, patients who had undergone radiotherapy, and children with open epiphyses. However, the single case of osteosarcoma reported among more than 250,000 patients treated with teriparatide in the U.S. and more than 300,000 patients treated worldwide, is within the expected background incidence of the tumor in the adult population (1 case in 250,000 patient-years)^35^. In the present investigation, all definitive operations were performed by a single surgeon and followed at the same department. Limitations of the study are the small sample size, short follow-up, and that no reliable and sensitive scores were used to assess the outcomes. We are aware that all the patients were different for type of fracture and surgical procedure performed, time from injury to administration of teriparatide and length of follow-up, but the administration of teriparatide seems to improve bone healing in problem fractures. Although the efficacy of teriparatide for delayed or non-unions is still unclear, it may induce an angiogenetic response which counteracts the pathological features responsible for development of bone union disorders. Further well-designed studies need to assess the efficacy of teriparatide in the setting of fracture nonunion.

## Figures and Tables

**Figure 10:**